# Mine ventilation system reliability evaluation based on a Markov chain

**DOI:** 10.1038/s41598-022-22098-z

**Published:** 2022-10-12

**Authors:** Li Liu, Jian Liu, Qichao Zhou

**Affiliations:** 1 College of Safety Science and Engineering, Liaoning Technical University, Huludao, 125105 Liaoning China; 2 Key Laboratory of Mine Thermo-Motive Disaster and Prevention, Ministry of Education, Huludao, 125105 Liaoning China

**Keywords:** Engineering, Mathematics and computing

## Abstract

Mine ventilation systems play a key role in creating and sustaining a healthy and safe working environment within the mine, and as such, should always be maintained at optimal performance levels. This paper establishes a model based on Markov chain that can quickly evaluate the reliability of the ventilation system. Firstly, the operation status of the ventilation system is divided into normal, risk and failure. Then, according to the failure rate and repair rate of the system, the operation state of the system under the specified total operation time *T* and time interval Δ*t* is simulated based on Monte Carlo method, the Markov chain state transfer probability matrix of the system can be obtained. Combined with the current operation state of the system, the reliability indexes such as the system operation state transfer probability and the steady state probability in the future can be quickly analyzed to realize the rapid evaluation of the operation reliability of the ventilation system. Finally, the model is used to evaluate the reliability of XQ mine ventilation system, which shows the effectiveness of the model. This research provides theoretical reference and technical support for mine safety production.

## Introduction

Mine safety is an important guarantee for mine production. Mine ventilation systems play a key role in creating and sustaining a healthy and safe working environment within the mine, and as such, should always be maintained at optimal performance levels. Real-time and rapid evaluation of ventilation system reliability can help us identify faults and concealed hazards in time, and provide a sound scientific basis for ventilation system design and transformation, so as to ensure the normal production of mines.

At present, the assessment of the reliability of the mine mainly focuses on the power system, transportation system and mechanical equipment. In order to improve the reliability of pump equipment for mine drainage, taking kimberlite mine as an example, ovchinnikov^1^ proposed a method to improve the service life of segmented pump, which has been fully confirmed. Ozdemir and kumral^2^ analyzed and explained the influence of human factors on the reliability of mining equipment, proposed a combined method based on reliability analysis, statistical reasoning and machine learning technology, and conducted a case study on transport trucks in mining operations. Krunic et al.^3^ proposed two model approaches to life cycle assessment of auxiliary mining machines: one based on reliability theory and the other based on cost–benefit principle, which provides decision basis for the effectiveness of further operation, maintenance or replacement of machinery. Allahkarami et al.^4^ studied the application of the mixed vulnerability model in the reliability analysis of mining equipment to describe the observed and unobserved heterogeneity, and used the field data of dump truck fleets in open-pit mines to evaluate the ability of the model. Stanek and venkata^5^ made a detailed analysis of the reliability of the mine power system, described the multifaceted effort to estimate the failure rates of equipment applicable to the mine environment, and used fault tree analysis to predict the probability of dangerous voltages on various mining equipment frames. Samanta et al.^6^ analyzed the reliability of the subsystems of the mining system: Mining scraper system, mine loader system and emergency equipment system. Barabady and kumar^7^ took the Jajarm bauxite crushing plant in Iran as an example to evaluate the reliability of mining and equipment. Some other studies have characterized the failure data of the loader / unloader^8^ and the longwall working face equipment^9^ by fitting the probability distribution with graphics and analysis techniques.

However, only a small number of researchers focus on the ventilation system, which is vital to mine safety production. Liu et al.^10^ evaluated the reliability of the mine roadway and proposed that the air volume and quality of each air path are affected by many factors, such as the main fan, the roadway wind resistance and pressure, the natural wind pressure, and the state of the ventilation structure. Therefore, it is required that the reliability of each air path must be calculated simultaneously with the ventilation network on the basis of comprehensively considering the above factors. Xu et al.^11^ proposed that structures can be divided into three types according to their functions: wind flow cutoff structures, wind flow passing structures and wind flow regulating structures. The reliability calculation methods of different structures are different, and with the increase of service time, the increase of wear will lead to the change of calculation methods of structures, which increases the difficulty of reliability research of structures. During the operation of the fan, the impeller will be subjected to the aerodynamic force caused by the huge centrifugal force, gravity and air flow in the casing, which will become the main cause of fan failure. Therefore, the research on the reliability of the fan is mainly focused on the impeller^12–14^. Cheng et al.^15^ divided the ventilation system into six subsystems: mine ventilation power subsystem, network structure and its mode subsystem, ventilation facilities subsystem, monitoring subsystem, disaster prevention facilities subsystem, and ventilation management subsystem. They comprehensively analyzed the technical capacity, work task, economy, complexity, importance and other aspects, and proposed a distribution model of ventilation system reliability based on AHP. Kumral^16^ plans a safety management system for the five basic operations in the production cycle of underground mines: drilling, blasting, loading, transportation / lifting and ventilation. While performing all subsystem functions at the lowest cost, the expected reliability and reliability estimation variance of the system are realized and solved by genetic algorithm (GA).

By analyzing the literature on reliability evaluation of mine ventilation system, there are two main approaches to study the reliability of mine ventilation systems: one is to analyze the reliability of ventilation system components, i.e., ventilation roads, ventilation power facilities and ventilation structures, and then determine the reliability of the entire ventilation system through simple mathematical operations. The other is to divide the ventilation system into a series of subsystems and consider that the failure of any one subsystem will lead to the failure of the ventilation system, and determine the reliability of the system as a whole by evaluating the reliability of the subsystems. Analysis of the existing methods for evaluating the reliability of mine ventilation systems reveals the following main problems: (1) The mine ventilation network is a complex hybrid system with multiple links, time-varying, nonlinear, and strong coupling characteristics, while the existing ventilation system reliability evaluation method adopts a local-to-whole analysis approach, treating the components of the mine ventilation system as independent individuals without mutual influence, which is at odds with reality, that is, the impact of structural dependence is ignored. (2) The computational cost of analysis method and numerical evaluation is high, which can not realize the real-time and rapid evaluation of ventilation system reliability. (3) The factors affecting the reliability of the ventilation system are complex and diverse, and the changes in the system state are uncertain, which are difficult to describe with existing models. (4) Ventilation system reliability analysis is mostly focused on the current ventilation system reliability judgment, which can only be used offline.

Therefore, a new method for reliability evaluation of ventilation systems that can solve the above problems is urgently sought. The ventilation system is a typical, repairable system^17^. The ventilation system operational state after the current period is related to the current state, but not to the previous state. In other words, the ventilation system has "no memory." The Markov chain is a model that describes the transition between states over time. It assumes that the future state only depends on the present state and has no relationship with the past state—i.e., a "memoryless process"^18^, and that, the future development process can be predicted given the current knowledge or information.

Therefore, this paper tries to start from the overall operation state of the system, grasp the change trend of the system from a macroscopic perspective, consider the operation process of the ventilation system as a Markov process, and evaluate the reliability of the ventilation system by using Markov chain. This method takes the whole system as the research object, which can avoid the disadvantage that it is difficult to consider the structural dependence among the components by the local-to-whole analysis approach, and the calculation method is simple, which can realize the real-time and rapid evaluation of the reliability of the ventilation system. The Markov chain has been applied to solve similar problems^19–23^and have been applied to more widely understood ventilation systems, such as indoor ventilation. In order to explore the impact of residents opening windows on indoor air flow, Fritsch et al.^24^ proposed a stochastic model using Markov chains to generate time series of window angles. Using this model in building air penetration design schemes can significantly improve its authenticity. Mei et al.^25^ developed a new high-order (second-order and third-order) Markov chain model based on weight factors to simulate indoor particle diffusion and deposition under fixed and dynamic ventilation modes. This model is expected to provide an alternative for the rapid prediction of indoor air particles (and gas) pollutants under transient flow. Huang et al.^26^ developed an improved Markov chain model based on which to predict transient particle transport and reduce the computational cost of predicting transient particle transport under periodic ventilation. Therefore, this paper attempts to evaluate the reliability of the mine ventilation system based on Markov chain, and achieve the purpose of quickly predicting the operating state of the ventilation system.

The main contribution of this paper focus on both theoretical value and practical applications. Its theoretical value can be concluded as the following four aspects. Firstly, the operation process of the ventilation system is regarded as a Markov process, and the reliability evaluation is transformed into a stochastic mathematical problem, which realizes the quantitative evaluation of the ventilation system reliability. Secondly, it avoids the disadvantage of ignoring the structure dependence in the previous reliability evaluation methods of mine ventilation system. Thirdly, real-time and rapid evaluation of ventilation system reliability is achieved. Finally, the prediction of the mine ventilation system reliability in the future period is realized. In terms of practical applications, the model provides guidance for provides guidance for system operation and maintenance, and can be applied to many repairable systems in the engineering field (such as power grid).

## Methods

### Markov chain theory

A Markov chain is a stochastic process that evaluate an event state and the transition law between states27. $E{ = }\left\{ {e_{1} ,e_{2} ,e_{3} ,....e_{n} } \right\}$ describes the discrete states, $T{ = }\left\{ {t_{1} ,t_{2} ,t_{3} ,....t_{n} } \right\}$ represents the discrete time of the event, and $\left\{ {\left. {X(t_{i} )} \right|i = 1,2,...,n} \right\} \in E$ is the event state at any time. The probability of the event in state $e_{n}$ at time tn can be defined by conditional probability as Eq. (1):

1 $$F_{{X(t_{n} )\left| {X(t_{1} )} \right....X(t_{n - 1} )}} = P\left[ {X(t_{n} ) = e_{n} \left| {X(t_{1} ) = e_{1} ...X(t_{n - 1} ) = e_{n - 1} } \right.} \right]$$

If:

2 $$F_{{X(t_{n} )\left| {X(t_{n - 1} )} \right.}} = F_{{X(t_{n} )\left| {X(t_{1} )...X(t_{n - 1} )} \right.}}$$

This relationship indicates that the process is "memoryless," so the discrete process is a Markov chain.

### Mine ventilation system characteristics

**Figure 1:**
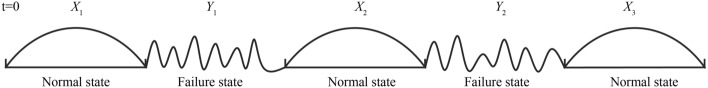
Ventilation system’s operational process (repairable system).

Mine ventilation systems are repairable systems—i.e., after system failure and subsequent maintenance, the system can re-enter the normal state, in which the working state and life of the system are the same as before the failure. Furthermore, the ventilation system’s operational state during the current period is related exclusively to the current state, and has no relationship with the previous state. Thus, the ventilation system can be considered to have "no memory." The operational process is shown in Fig. 1. *X*_*i*_ and *Y*_*i*_ represent the normal working state and fault maintenance state, respectively, of the i-th cycle in the system operational process. In addition, the ventilation system’s operational state is dynamic and random—i.e., when the system is in normal state (*X*_*i*_), fault state (*Y*_*i*_) cannot be determined. To sum up, the ventilation system’s operational process can be regarded as a Markov Chain, and the ventilation system reliability evaluation problem can be transformed into a random probability problem.

**Figure 2:**
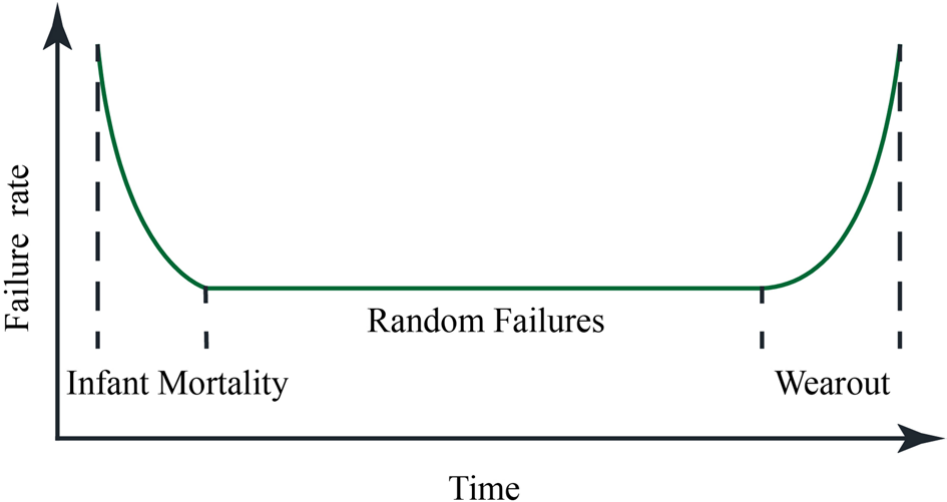
Failure rate "Bathtub curve".

In practical engineering, there are complex correlations among failure modes of mine ventilation system structures, so the specific probability distribution of variables is difficult to obtain and expensive. As such, the following assumptions should be made when evaluating the ventilation system’s reliability based on a Markov chain:

1. In theory, the system’s common life distribution mainly includes exponential, Poisson, normal, and lognormal distribution, etc. Because the random variable values in exponential distribution are all nonnegative real numbers, exponential distribution is often used to describe the life distribution of various systems, equipment, and components—e.g., the life of electronic components and the service time in a random service system^28^. Exponential distribution is the only "memoryless" function, and is widely used in reliability evaluation. Therefore, herein, we assume that the ventilation system life distribution is an exponential distribution.
2. Maintenance cycles vary from mine to mine. For example, the structures in mines that are deep, contain a high salt concentration, and/or exhibit high humidity tend to seriously corrode; as such, the maintenance cycle is only a few months. In contrast, dry and high altitude mine maintenance cycles can reach several years or more. *R*_*F*_ is the threshold for determining whether the ventilation system is in the failure state—and failure is indicated when the reliability is less than *R*_*F*_ (*R* < *R*_*F*_). When the same mine is undergoing different life cycles, the failure rate and maintenance rate differ. Estimation of failure rates and maintenance rate provides a key input to risk and reliability quantification^29^, and the failure rate distribution can be approximated as a "bathtub curve" (Fig. 2).

**Figure 3:**
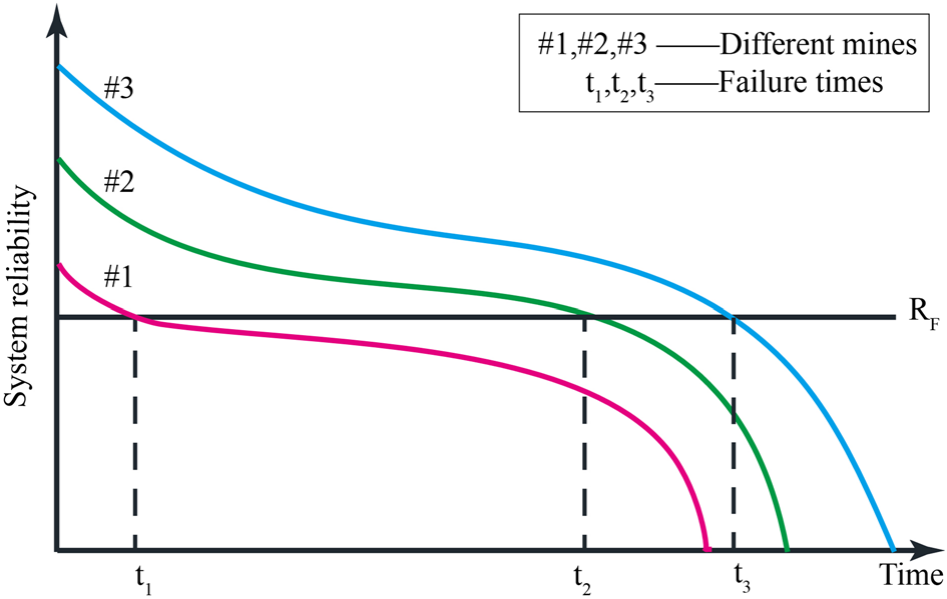
Failure models of different mines.

Estimation of the probability of accident occurrence is equally as important as consequence analysis in order to estimate the risk of the system, thus to make a risk-based decision^30^. In practical engineering, the failure rate is a function of time. It is relatively high during infant mortality, a consequence of engineering errors, such as inadequate ventilation system design and manufacturing, or incorrect construction^31^. During the random failures period, the failure rate of the system remains basically unchanged and whatever system failure occurs is mainly caused by accidental factors, which is the critical period for us to evaluate the system reliability. In the wear out period, the failure rate will increase due to aging and wear and tear on structures, such as roadways and fans, etc. Moreover, differences in failure type and degree, the technical level of maintenance technicians, and numerous other factors will also lead to different maintenance rates. Considering these potential variations, the failure models of different mines are shown in Fig. 3.

The random failure period is the effective period of system reliability evaluation. In the random failure period, the failure rate $\lambda$ is low and stable. In addition, the main purpose of this paper is to introduce the applicability of Markov model to evaluate the reliability of mine ventilation system. The constant failure rate model (CFR) is the simplest case in system reliability evaluation, which can greatly reduce the calculation amount and time. Therefore, this study is based on the CFR model, that is $\lambda (t) = \lambda$. In the production process of the same mine, except for catastrophic accidents such as explosion and fire, other fault types and fault degrees are almost the same, and the level of maintenance personnel is also roughly the same, resulting in no great difference in maintenance time. Based on this, it is assumed that the maintenance rate is constant, that is $\mu (t) = \mu$. In future research projects, we plan to consider the time-dependent failure rate and maintenance rate, in order to obtain results closer to reality.

Based on the above assumptions, the ventilation system’s life distribution function (i.e., the probability that the system life does not exceed *t*) can be obtained as Eq. (3):

3 $$F(t) = P(T \le t) = 1 - e^{ - \lambda t} (t > 0)$$

The repair time distribution function after system failure is as Eq. (4):

4 $$G(t) = P(\tau \le t) = 1 - e^{ - \mu t} (t > 0)$$

And:

5 $$\lambda { = }\frac{1}{{{\text{MTBF}}}},\mu { = }\frac{1}{{{\text{MTTR}}}}$$

where *T* is the ventilation system life span; $\tau$ is the time from ventilation failure to maintenance; MTBF is the mean time between failure—for repairable systems, refers to the working time between two adjacent failures, namely maintenance cycle; MTTR is the mean time to repair— refers to the average time for each repairable system failure.

### Mine ventilation system reliability evaluation model

When constructing the reliability evaluation model of mine ventilation system based on Markov chain, firstly, the operation state of the system is divided; then, the Markov state transfer probability matrix of the mine ventilation system is obtained based on the operating states that the system is in at different time periods and the transitions of the operating states of the system in adjacent time periods. Given that the initial state of the ventilation system is known, the current operating state of the system and the change trend of the system operating state in the future period can be quickly obtained.

1. Ventilation system’s operational state categories

The ventilation system’s reliability analysis is mostly based on the two-state hypothesis (fault state or normal state). However, due to its complex structure, the changeable environment, the wear and aging of parts, and the roadway’s deformation in the operational process, the ventilation system may appear in one or more intermediate states. As such, the ventilation system is a polymorphic system. Thus, to evaluate the system reliability more accurately, the system state must be defined more specifically, which requires additional research. According to the mine ventilation system’s main parameters during the operational process—i.e., air quality and air volume, the operational states of ventilation equipment and structures, and the time required for fault maintenance—the ventilation system’s operational states are divided into three types:

1. State 1 (normal state) *e*_1_: There is no fault in the ventilation system. All the equipment and their components are in good working condition, and each parameter is within the specified range.
2. State 2 (risk state) *e*_2_: Some constraints in the system cannot be satisfied, but the system will not cause large-scale damage after putting in standby equipment or performing readjustments. Examples of potential risk state issues consist of door damage, roadway deformation, temporary blockage of transport vehicles, fan blade wear or damage, accidental air leakage, and natural wind pressure in summer, etc.
3. State 3 (fault state) *e*_3_: There is a fault in the ventilation system that causes a certain degree of casualties or property loss. In this scenario, wide range, long-term adjustments are required to restore the safe and stable operational state of the system. Examples of potential fault state issues include spalling roof fall, unexpected air door opening and long-term failure to recover, complete destruction of the air door, and serious blockage of the roadway, etc.

(2) State transition and transition probability matrix.

**Figure 4:**
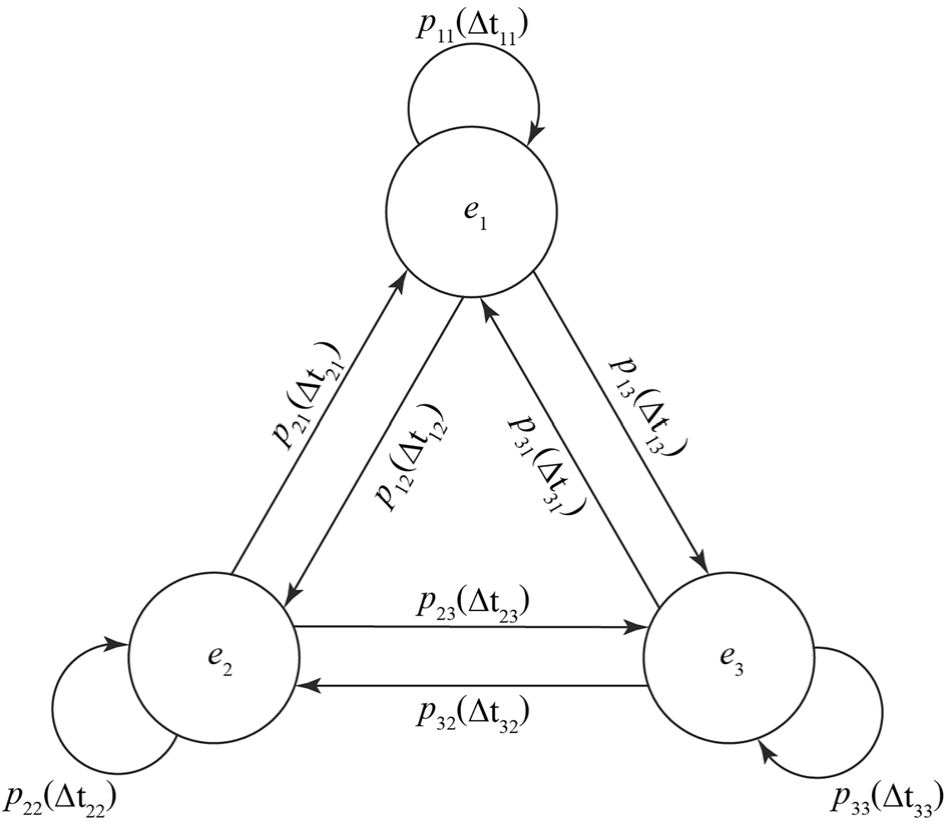
Ventilation system state transition schematic diagram.

Markov chains with finite discrete states can be represented by digraphs. Nodes represent states, edges represent transitions between states, and values on edges represent transition probabilities. According to the probability defined on the directed edge, a sequence of states can be generated from an initial state by randomly transferring between states^32^. The ventilation system’s three operational states are expressed as ***E*** = {*e*_1_, *e*_2_, *e*_3_}. The ventilation system’s probability of changing from state *i* to state *j* is set as *P*_*ij*_ in the time interval Δ*t*_*ij*_, *i* is the initial state and *j* is the state after transition. Thus, the probability of state 1 changing to state 2 is *P*_12_, and the probability of state 1 changing to state 3 is *P*_13_. The whole system’s state transition process is shown in Fig. 4.

The whole ventilation system’s state transition is expressed in the form of matrix ***P***:

6 $${\varvec{P}}{ = }\left[ \begin{gathered} p_{11} (\Delta t_{11} ){\kern 1pt} {\kern 1pt} {\kern 1pt} {\kern 1pt} {\kern 1pt} {\kern 1pt} {\kern 1pt} {\kern 1pt} {\kern 1pt} {\kern 1pt} {\kern 1pt} {\kern 1pt} p_{12} (\Delta t_{12} ){\kern 1pt} {\kern 1pt} {\kern 1pt} {\kern 1pt} {\kern 1pt} {\kern 1pt} {\kern 1pt} {\kern 1pt} {\kern 1pt} {\kern 1pt} {\kern 1pt} {\kern 1pt} p_{13} (\Delta t_{13} ){\kern 1pt} \hfill \\ p_{21} (\Delta t_{21} ){\kern 1pt} {\kern 1pt} {\kern 1pt} {\kern 1pt} {\kern 1pt} {\kern 1pt} {\kern 1pt} {\kern 1pt} {\kern 1pt} {\kern 1pt} {\kern 1pt} {\kern 1pt} p_{22} (\Delta t_{22} ){\kern 1pt} {\kern 1pt} {\kern 1pt} {\kern 1pt} {\kern 1pt} {\kern 1pt} {\kern 1pt} {\kern 1pt} {\kern 1pt} {\kern 1pt} {\kern 1pt} p_{23} (\Delta t_{23} ) \hfill \\ p_{31} (\Delta t_{31} ){\kern 1pt} {\kern 1pt} {\kern 1pt} {\kern 1pt} {\kern 1pt} {\kern 1pt} {\kern 1pt} {\kern 1pt} {\kern 1pt} {\kern 1pt} {\kern 1pt} {\kern 1pt} p_{32} (\Delta t_{32} ){\kern 1pt} {\kern 1pt} {\kern 1pt} {\kern 1pt} {\kern 1pt} {\kern 1pt} {\kern 1pt} {\kern 1pt} {\kern 1pt} {\kern 1pt} {\kern 1pt} p_{33} (\Delta t_{33} ){\kern 1pt} {\kern 1pt} {\kern 1pt} \hfill \\ \end{gathered} \right]$$

and meets the following requirements:

7 $$\left\{ \begin{gathered} p_{ij} (\Delta t_{ij} ) \ge 0,{\kern 1pt} {\kern 1pt} {\kern 1pt} {\kern 1pt} {\kern 1pt} {\kern 1pt} {\kern 1pt} {\kern 1pt} {\kern 1pt} {\kern 1pt} i,j = 1,2,3 \hfill \\ \sum\limits_{j = 1}^{3} {p_{ij} (\Delta t_{ij} ) = 1,{\kern 1pt} {\kern 1pt} {\kern 1pt} {\kern 1pt} {\kern 1pt} {\kern 1pt} {\kern 1pt} {\kern 1pt} {\kern 1pt} i = 1,2,3} \hfill \\ \end{gathered} \right.$$

It is assumed that the ventilation system’s state change stochastic process is a time homogeneous Markov chain^33^:

8 $$p\left[ {X(t + \Delta t) = e_{j} \left| {X(t) = e_{i} } \right.{\kern 1pt} } \right]{\kern 1pt} = p\left[ {X(\Delta t) = e_{j} \left| {X(0) = e_{i} } \right.{\kern 1pt} } \right] = p_{ij} (\Delta t)$$

If the time interval $\Delta t$ of each state transition is the same, the ventilation system’s state transition probability matrix can be simplified as Eq. (9):

9 $${\varvec{P}}{ = }\left[ \begin{gathered} p_{11} {\kern 1pt} {\kern 1pt} {\kern 1pt} {\kern 1pt} {\kern 1pt} {\kern 1pt} {\kern 1pt} {\kern 1pt} {\kern 1pt} {\kern 1pt} {\kern 1pt} {\kern 1pt} p_{12} {\kern 1pt} {\kern 1pt} {\kern 1pt} {\kern 1pt} {\kern 1pt} {\kern 1pt} {\kern 1pt} {\kern 1pt} {\kern 1pt} {\kern 1pt} {\kern 1pt} p_{13} \hfill \\ p_{21} {\kern 1pt} {\kern 1pt} {\kern 1pt} {\kern 1pt} {\kern 1pt} {\kern 1pt} {\kern 1pt} {\kern 1pt} {\kern 1pt} {\kern 1pt} {\kern 1pt} {\kern 1pt} p_{22} {\kern 1pt} {\kern 1pt} {\kern 1pt} {\kern 1pt} {\kern 1pt} {\kern 1pt} {\kern 1pt} {\kern 1pt} {\kern 1pt} {\kern 1pt} p_{23} \hfill \\ p_{31} {\kern 1pt} {\kern 1pt} {\kern 1pt} {\kern 1pt} {\kern 1pt} {\kern 1pt} {\kern 1pt} {\kern 1pt} {\kern 1pt} {\kern 1pt} {\kern 1pt} {\kern 1pt} p_{32} {\kern 1pt} {\kern 1pt} {\kern 1pt} {\kern 1pt} {\kern 1pt} {\kern 1pt} {\kern 1pt} {\kern 1pt} {\kern 1pt} {\kern 1pt} p_{33} {\kern 1pt} {\kern 1pt} {\kern 1pt} \hfill \\ \end{gathered} \right]$$

The state transition probability matrix of this study is obtained through statistics. According to the law of large numbers, the more samples, the closer the statistical probability is to the real probability distribution. Therefore, when the number of samples is sufficient, the state transition probability can be determined according to the proportion of samples transferred between states *n*_*ij*_ in the total sample pool. Subsequently, the ventilation system’s state transition probability matrix can be obtained. When the number of samples tends to infinity, the statistical value of the state transition probability matrix infinitely approaches the real value. Statistics for the state transition number of samples are shown in Table 1.

**Table 1:** Sample number of state transition.

Transition state Initial state	*e*_1_	*e*_2_	*e*_3_	Total
*e*_1_	*n*_11_	*n*_12_	*n*_13_	*N*_1_
*e*_2_	*n*_21_	*n*_22_	*n*_23_	*N*_2_
*e*_3_	*n*_31_	*n*_32_	*n*_33_	*N*_3_

The state transition probability matrix is obtained by:

$${\varvec{P}}{ = }\left[ \begin{gathered} p_{11} {\kern 1pt} {\kern 1pt} {\kern 1pt} {\kern 1pt} {\kern 1pt} {\kern 1pt} {\kern 1pt} {\kern 1pt} {\kern 1pt} {\kern 1pt} {\kern 1pt} {\kern 1pt} p_{12} {\kern 1pt} {\kern 1pt} {\kern 1pt} {\kern 1pt} {\kern 1pt} {\kern 1pt} {\kern 1pt} {\kern 1pt} {\kern 1pt} {\kern 1pt} {\kern 1pt} p_{13} \hfill \\ p_{21} {\kern 1pt} {\kern 1pt} {\kern 1pt} {\kern 1pt} {\kern 1pt} {\kern 1pt} {\kern 1pt} {\kern 1pt} {\kern 1pt} {\kern 1pt} {\kern 1pt} {\kern 1pt} p_{22} {\kern 1pt} {\kern 1pt} {\kern 1pt} {\kern 1pt} {\kern 1pt} {\kern 1pt} {\kern 1pt} {\kern 1pt} {\kern 1pt} {\kern 1pt} p_{23} \hfill \\ p_{31} {\kern 1pt} {\kern 1pt} {\kern 1pt} {\kern 1pt} {\kern 1pt} {\kern 1pt} {\kern 1pt} {\kern 1pt} {\kern 1pt} {\kern 1pt} {\kern 1pt} {\kern 1pt} p_{32} {\kern 1pt} {\kern 1pt} {\kern 1pt} {\kern 1pt} {\kern 1pt} {\kern 1pt} {\kern 1pt} {\kern 1pt} {\kern 1pt} {\kern 1pt} p_{33} {\kern 1pt} {\kern 1pt} {\kern 1pt} \hfill \\ \end{gathered} \right]{ = }\left[ \begin{gathered} {{n_{11} } \mathord{\left/ {\vphantom {{n_{11} } {N_{1} }}} \right. \kern-\nulldelimiterspace} {N_{1} }}{\kern 1pt} {\kern 1pt} {\kern 1pt} {\kern 1pt} {\kern 1pt} {\kern 1pt} {\kern 1pt} {\kern 1pt} {\kern 1pt} {\kern 1pt} {\kern 1pt} {\kern 1pt} {{n_{12} } \mathord{\left/ {\vphantom {{n_{12} } {N_{1} }}} \right. \kern-\nulldelimiterspace} {N_{1} }}{\kern 1pt} {\kern 1pt} {\kern 1pt} {\kern 1pt} {\kern 1pt} {\kern 1pt} {\kern 1pt} {\kern 1pt} {\kern 1pt} {\kern 1pt} {\kern 1pt} {{n_{13} } \mathord{\left/ {\vphantom {{n_{13} } {N_{1} }}} \right. \kern-\nulldelimiterspace} {N_{1} }} \hfill \\ {{n_{21} } \mathord{\left/ {\vphantom {{n_{21} } {N_{2} }}} \right. \kern-\nulldelimiterspace} {N_{2} }}{\kern 1pt} {\kern 1pt} {\kern 1pt} {\kern 1pt} {\kern 1pt} {\kern 1pt} {\kern 1pt} {\kern 1pt} {\kern 1pt} {\kern 1pt} {\kern 1pt} {{n_{22} } \mathord{\left/ {\vphantom {{n_{22} } {N_{2} }}} \right. \kern-\nulldelimiterspace} {N_{2} }}{\kern 1pt} {\kern 1pt} {\kern 1pt} {\kern 1pt} {\kern 1pt} {\kern 1pt} {\kern 1pt} {\kern 1pt} {\kern 1pt} {\kern 1pt} {{n_{23} } \mathord{\left/ {\vphantom {{n_{23} } {N_{2} }}} \right. \kern-\nulldelimiterspace} {N_{2} }} \hfill \\ {{n_{31} } \mathord{\left/ {\vphantom {{n_{31} } {N_{3} }}} \right. \kern-\nulldelimiterspace} {N_{3} }}{\kern 1pt} {\kern 1pt} {\kern 1pt} {\kern 1pt} {\kern 1pt} {\kern 1pt} {\kern 1pt} {\kern 1pt} {\kern 1pt} {\kern 1pt} {\kern 1pt} {\kern 1pt} {{n_{32} } \mathord{\left/ {\vphantom {{n_{32} } {N_{3} }}} \right. \kern-\nulldelimiterspace} {N_{3} }}{\kern 1pt} {\kern 1pt} {\kern 1pt} {\kern 1pt} {\kern 1pt} {\kern 1pt} {\kern 1pt} {\kern 1pt} {\kern 1pt} {\kern 1pt} {{n_{33} } \mathord{\left/ {\vphantom {{n_{33} } {N_{3} }}} \right. \kern-\nulldelimiterspace} {N_{3} }}{\kern 1pt} {\kern 1pt} {\kern 1pt} \hfill \\ \end{gathered} \right]$$

#### Stationary state probability of ventilation system

Assuming that the initial ventilation system state is ${\varvec{\phi}}(0)$, according to the definition of transition probability matrix ***P***, after Δ*t* time, the ventilation system’s state distribution is as follows:

10 $${\varvec{\phi}}(1) = {\varvec{\phi}}(0){\varvec{P}}$$

After *m*Δ*t*, the ventilation system’s state distribution is as follows:

11 $${\varvec{\phi}}(m) = {\varvec{\phi}}(m - {1}){\varvec{P}}{ = }{\varvec{\phi}}({0}){\varvec{P}}^{m}$$

When the number of time intervals is *m* → ∞, the ventilation system’s state gradually approaches a certain stable value, which is called the stationary state probability or long-term state probability.

12 $${\varvec{\phi}}(\infty ) = \mathop {\lim }\limits_{m \to \infty } {\varvec{\phi}}(n){ = }\mathop {\lim }\limits_{m \to \infty } {\varvec{\phi}}({0}){\varvec{P}}^{m}$$

13 $${\varvec{\phi}}(\infty ) \cdot P = {\varvec{\phi}}(\infty )$$

The stationary state probability means that regardless of the ventilation system’s initial state, after a long enough time, the probability of being in state *i* will be close to *π*_*i*_(∞), and:

14 $$\sum\limits_{i = 1}^{3} {\pi_{i} (\infty ) = 1}$$

## Case study and results analysis

### Overview of XQ mine ventilation system

**Figure 5:**
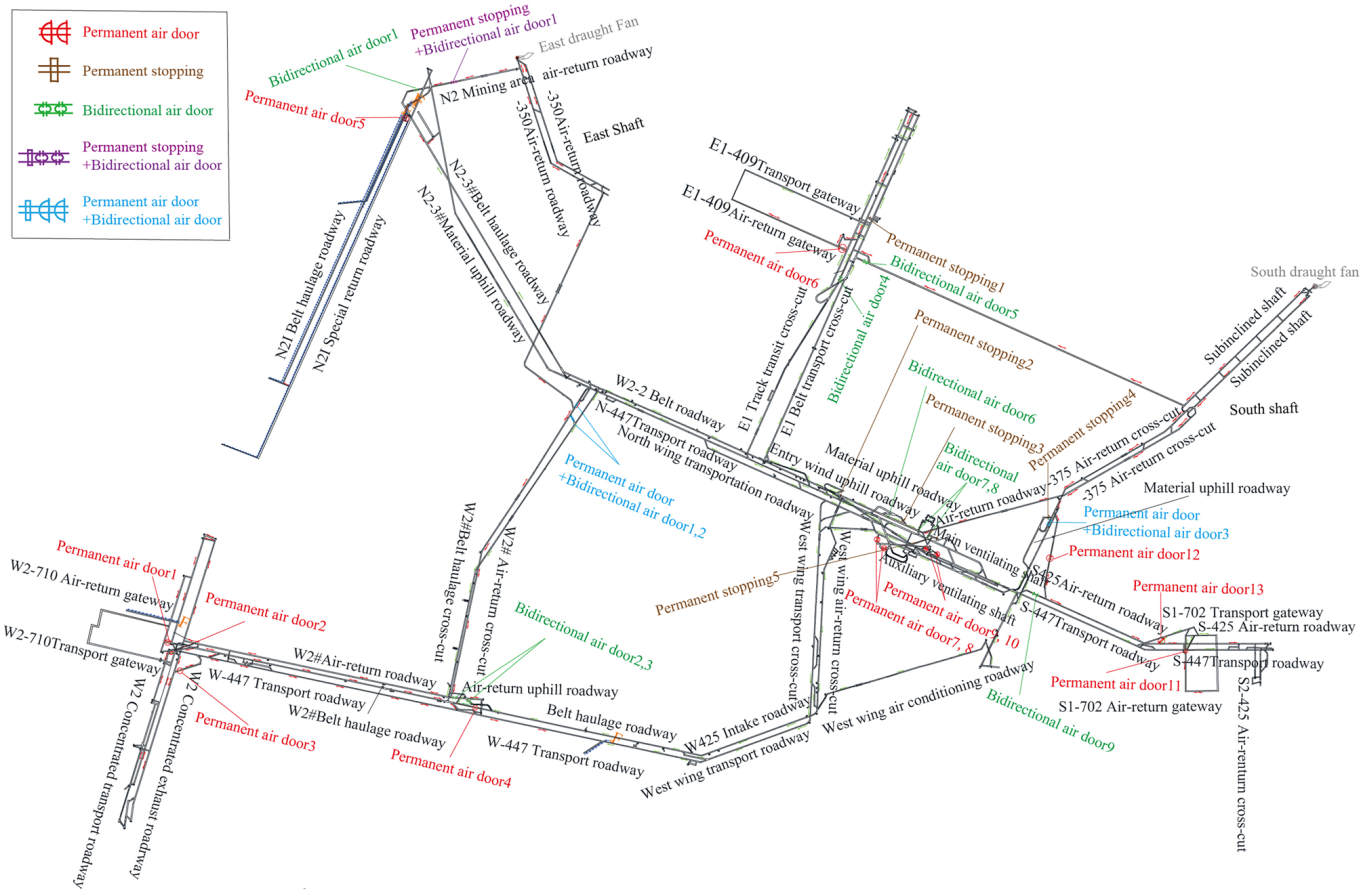
XQ mine System diagram.

As an example, production mine XQ’s reliability was evaluated using the method proposed herein. The system diagram is shown in Fig. 5. XQ mine has four shafts: main shaft, auxiliary shaft, east shaft and south shaft. The main shaft lifts raw coal, the auxiliary shaft lifting gangue, materials and personnel, and the east and south shafts exhaust air. The fan models of the east and south air shafts are: BD-II-8-No24, GAF21.1-12.6-1. The total air intake is 9341 m^3^/min.

The maintenance cycle and average repair time of the structures, roadways, and ventilation power facilities in the mine are shown in Table 2. MTBF and MTTR are given according to the numbering sequence of various structures, roadways and fans in Fig. 5.

**Table 2:** XQ mine maintenance cycle and repair time data.

Types		Number	MTBF/year	MTTR/day
Structures	Permanent air door	13	7,8,6,5,11,8,10,10,10,10,1,3,10	All for 1 day
	Bidirectional air door	9	10,10,10,2,2,5,3,10,11	All for 1 day
	Permanent stopping	5	All for 20 years	All for 1 day
	Permanent air door + Bidirectional air door	3	All for 3 years	All for 1 day
	Permanent stopping + Bidirectional air door	1	10	2
Roadways	Semicircular arch bolting and shotcreting air-return roadway	14	20,3,5,10,10,10,10,15,1,10,20,10,10,10	20,12,22,23,29,11,26,24,28,12,23,29,16,23
	Semicircular arch bolting and shotcreting belt roadway	6	5,3,3,20,10,10	28,19,25,27,13,9
	Semicircular arch U-shaped shed track roadway	6	20,10,1,20,10,10	36,20,25,37,21,36
	Semicircular arch U-shaped shed belt cross-cut, Track uphill roadway, Track roadway, Rectangular/Trapezoidal shed air-return cross-cut	10	7,5,7,6,7,7,5,7,5,7	2,14,12,11,12,8,10,12,15,11
	Semicircular arch brickwork belt roadway, Track roadway, Air-return uphill roadway, Air-return roadway	8	All for 9 years	All for 30 days
	Semicircular arch bolting and shotcreting track uphill roadway, Track cross-cut, Belt uphill roadway	5	All for 10 years	42,60,36,20,27
	Rectangular bolting with wire mesh belt roadway, Air-return roadway, Track roadway	5	All for 12 years	8,13,5,13,7
	Rectangular bolting with wire mesh belt gateway, Track gateway	3	All for 2 years	All for 1 day
	Rectangular/Trapezoidal shed belt roadway	5	10,5,10,7,7	23,21,19,17,28
Ventilation power facilities	East shaft	1	no fault	–
	South shaft	1	no fault	–

### Reliability evaluation of XQ mine ventilation system

The number of system state transition samples monitored by sensors is limited, which leads to the lack of accumulation of actual operation data, and it is difficult to obtain the state transition probability matrix of the system. Therefore, the Sequential Monte Carlo method was used to simulate the ventilation network’s operational state. According to Eq. (5), the ventilation system’s failure rate and repair rate are calculated by day:

$$\begin{aligned} \overline{{{MTBF}}} &{ = }\frac{{\left( {\sum\limits_{i = 1}^{31} {{MTBF}_{structures} + \sum\limits_{i = 1}^{{{62}}} {{MTBF}_{ roadway} + } \sum\limits_{i = 1}^{2} {{MTBF}_{fan} } } } \right)}}{{31{ + 62}}} \hfill \\ & { = 9(}{year}{)} \hfill \\ \end{aligned}$$

$$\begin{aligned} \overline{{{MTTR}}} & { = }\frac{{\left( {\sum\limits_{i = 1}^{31} {{MTTR}_{structures} + \sum\limits_{i = 1}^{{{62}}} {{MTTR}_{ roadway} + } \sum\limits_{i = 1}^{2} {{MTTR}_{fan} } } } \right)}}{{31{ + 62}}} \hfill \\ & { = 14}{\text{.14(}}{day}{)} \hfill \\ \end{aligned}$$

$$\lambda { = }\frac{1}{{\overline{{{MTBF}}} \times 365}} = 0.0003{0}4$$

$$\mu { = }\frac{1}{{\overline{{{MTTR}}} }} = 0.07{1}$$

The time interval $\Delta t$ was set as one day, the ventilation system’s failure probability in time interval $\Delta t$ was $\lambda { = }0.0003{0}4$, and the maintenance rate was $\mu { = }0.07{1}$. A total of 8000 simulations were performed, with 365 $\Delta t$ for each simulation—i.e., the total duration *T* = 365 days. The number of transfer samples between each state is shown in Table 3.

**Table 3:** State transition data.

Initial state	Transition state			Total
	*e*_1_	*e*_2_	*e*_3_	
*e*_1_	1,660,429	80,769	491	1,741,689
*e*_2_	80,986	1,080,924	23	1,161,933
*e*_3_	491	24	6505	7020
Total	1,741,906	1,161,717	7019	2,910,642

The ventilation system’s state transition probability matrix was obtained:

$$P = \left( {\begin{array}{*{20}r} \hfill {0.953344} & \hfill {0.046374} & \hfill {0.000282} \\ \hfill {0.069699} & \hfill {0.930281} & \hfill {0.000020} \\ \hfill {0.069943} & \hfill {0.003419} & \hfill {0.926638} \\ \end{array} } \right)$$

According to the state transition matrix ***P***…

- If the system is currently in normal state *e*_1_, the probability that after time interval $\Delta t$ it will remain in normal state *e*_1_, be transferred to risk state *e*_2_, or be transferred to fault state *e*_3_ is 95.3344%, 4.6374%, and 0.0282%, respectively.
- For a system that is currently in risk state e2, the probability that after time interval $\Delta t$ it will remain in risk state *e*_2_, transfer to normal state *e*_1_, or transfer to the fault state *e*_3_ is 93.0281%, 6.9699%, and 0.0020%, respectively.
- For a system that is currently in fault state *e*_3_, the probability that after time interval $\Delta t$ it will remain in fault state *e*_3_, transfer to the normal state *e*_1_, or transfer to the risk state *e*_2_ is 92.6638%, 6.9943%, and 0.3419%, respectively.

Subsequently, assuming the initial state of the ventilation system is normal state—${\varvec{\phi}}(0){ = (1 0 0)}$, the value of ${\varvec{\phi}}(1) - {\varvec{\phi}}(365)$, i.e., the probability of the system being in states *e*_1_, *e*_2_, and *e*_3_ every day in a year, can be obtained quickly after 365 times of matrix multiplication, which takes only 0.188 s. The calculation process is as follows:

$$\begin{aligned} \phi (1) & = \phi (0) \cdot {\varvec{P}} \hfill \\ & = \left( {1{\kern 1pt} {\kern 1pt} {\kern 1pt} {\kern 1pt} {\kern 1pt} {\kern 1pt} {\kern 1pt} {\kern 1pt} {\kern 1pt} {\kern 1pt} {\kern 1pt} {\kern 1pt} {\kern 1pt} {\kern 1pt} 0{\kern 1pt} {\kern 1pt} {\kern 1pt} {\kern 1pt} {\kern 1pt} {\kern 1pt} {\kern 1pt} {\kern 1pt} {\kern 1pt} {\kern 1pt} {\kern 1pt} {\kern 1pt} {\kern 1pt} 0} \right)\left( {\begin{array}{*{20}r} \hfill {0.953344} & \hfill {0.046374} & \hfill {0.000282} \\ \hfill {0.069699} & \hfill {0.930281} & \hfill {0.000020} \\ \hfill {0.069943} & \hfill {0.003419} & \hfill {0.926638} \\ \end{array} } \right) \hfill \\ {\kern 1pt} {\kern 1pt} {\kern 1pt} {\kern 1pt} {\kern 1pt} {\kern 1pt} {\kern 1pt} {\kern 1pt} {\kern 1pt} {\kern 1pt} {\kern 1pt} {\kern 1pt} {\kern 1pt} {\kern 1pt} {\kern 1pt} {\kern 1pt} {\kern 1pt} {\kern 1pt} & = \left( {0.953344{\kern 1pt} {\kern 1pt} {\kern 1pt} {\kern 1pt} {\kern 1pt} 0.046374{\kern 1pt} {\kern 1pt} {\kern 1pt} {\kern 1pt} {\kern 1pt} {\kern 1pt} 0.000282} \right) \hfill \\ \end{aligned}$$

$$\begin{gathered} \phi (2) = \phi (0) \cdot \varvec{P}^{{2}} \hfill \\ {\kern 1pt} {\kern 1pt} {\kern 1pt} {\kern 1pt} {\kern 1pt} {\kern 1pt} {\kern 1pt} {\kern 1pt} {\kern 1pt} {\kern 1pt} {\kern 1pt} {\kern 1pt} {\kern 1pt} {\kern 1pt} {\kern 1pt} {\kern 1pt} {\kern 1pt} {\kern 1pt} = \left( {1{\kern 1pt} {\kern 1pt} {\kern 1pt} {\kern 1pt} {\kern 1pt} {\kern 1pt} {\kern 1pt} {\kern 1pt} {\kern 1pt} {\kern 1pt} 0{\kern 1pt} {\kern 1pt} {\kern 1pt} {\kern 1pt} {\kern 1pt} {\kern 1pt} {\kern 1pt} {\kern 1pt} {\kern 1pt} {\kern 1pt} 0} \right)\left( {\begin{array}{*{20}r} \hfill {0.953344} & \hfill {0.046374} & \hfill {0.000282} \\ \hfill {0.069699} & \hfill {0.930281} & \hfill {0.000020} \\ \hfill {0.069943} & \hfill {0.003419} & \hfill {0.926638} \\ \end{array} } \right)^{2} \hfill \\ {\kern 1pt} {\kern 1pt} {\kern 1pt} {\kern 1pt} {\kern 1pt} {\kern 1pt} {\kern 1pt} {\kern 1pt} {\kern 1pt} {\kern 1pt} {\kern 1pt} {\kern 1pt} {\kern 1pt} {\kern 1pt} {\kern 1pt} {\kern 1pt} {\kern 1pt} {\kern 1pt} = \left( {0.912117{\kern 1pt} {\kern 1pt} {\kern 1pt} {\kern 1pt} {\kern 1pt} {\kern 1pt} {\kern 1pt} {\kern 1pt} {\kern 1pt} {\kern 1pt} {\kern 1pt} {\kern 1pt} {\kern 1pt} 0.087352{\kern 1pt} {\kern 1pt} {\kern 1pt} {\kern 1pt} {\kern 1pt} {\kern 1pt} {\kern 1pt} {\kern 1pt} {\kern 1pt} {\kern 1pt} {\kern 1pt} {\kern 1pt} 0.000531} \right) \hfill \\ \,\,\,\,\,\,\,\,\,\,\,\,\,\,\,\,\,\,\,\,\,\,\,\,\,\,\,\,\,\,\,\,\,\,\,\,\,\,\,\,\,\,\,\,\,\,\,\,\, \vdots \hfill \\ \end{gathered}$$

$$\begin{gathered} \phi (365) = \phi (0) \cdot {\varvec{P}}^{{{365}}} \hfill \\ {\kern 1pt} {\kern 1pt} {\kern 1pt} {\kern 1pt} {\kern 1pt} {\kern 1pt} {\kern 1pt} {\kern 1pt} {\kern 1pt} {\kern 1pt} {\kern 1pt} {\kern 1pt} {\kern 1pt} {\kern 1pt} {\kern 1pt} {\kern 1pt} {\kern 1pt} {\kern 1pt} {\kern 1pt} {\kern 1pt} {\kern 1pt} {\kern 1pt} {\kern 1pt} {\kern 1pt} {\kern 1pt} {\kern 1pt} {\kern 1pt} = \left( {1{\kern 1pt} {\kern 1pt} {\kern 1pt} {\kern 1pt} {\kern 1pt} {\kern 1pt} {\kern 1pt} {\kern 1pt} {\kern 1pt} {\kern 1pt} 0{\kern 1pt} {\kern 1pt} {\kern 1pt} {\kern 1pt} {\kern 1pt} {\kern 1pt} {\kern 1pt} {\kern 1pt} {\kern 1pt} {\kern 1pt} 0} \right)\left( {\begin{array}{*{20}r} \hfill {0.953344} & \hfill {0.046374} & \hfill {0.000282} \\ \hfill {0.069699} & \hfill {0.930281} & \hfill {0.000020} \\ \hfill {0.069943} & \hfill {0.003419} & \hfill {0.926638} \\ \end{array} } \right)^{365} \hfill \\ {\kern 1pt} {\kern 1pt} {\kern 1pt} {\kern 1pt} {\kern 1pt} {\kern 1pt} {\kern 1pt} {\kern 1pt} {\kern 1pt} {\kern 1pt} {\kern 1pt} {\kern 1pt} {\kern 1pt} {\kern 1pt} {\kern 1pt} {\kern 1pt} {\kern 1pt} {\kern 1pt} {\kern 1pt} {\kern 1pt} {\kern 1pt} {\kern 1pt} {\kern 1pt} {\kern 1pt} {\kern 1pt} {\kern 1pt} {\kern 1pt} = \left( {0.599025{\kern 1pt} {\kern 1pt} {\kern 1pt} {\kern 1pt} {\kern 1pt} {\kern 1pt} {\kern 1pt} {\kern 1pt} {\kern 1pt} 0.398563{\kern 1pt} {\kern 1pt} {\kern 1pt} {\kern 1pt} {\kern 1pt} {\kern 1pt} {\kern 1pt} {\kern 1pt} {\kern 1pt} 0.002411} \right) \hfill \\ \end{gathered}$$

The ventilation system’s stationary state probability analytic value is obtained as follows:

$$\pi_{i} (\infty ) = [0.599025{\kern 1pt} {\kern 1pt} {\kern 1pt} {\kern 1pt} {\kern 1pt} {\kern 1pt} {\kern 1pt} {\kern 1pt} {\kern 1pt} 0.398563{\kern 1pt} {\kern 1pt} {\kern 1pt} {\kern 1pt} {\kern 1pt} {\kern 1pt} {\kern 1pt} {\kern 1pt} {\kern 1pt} 0.002411]$$

**Figure 6:**
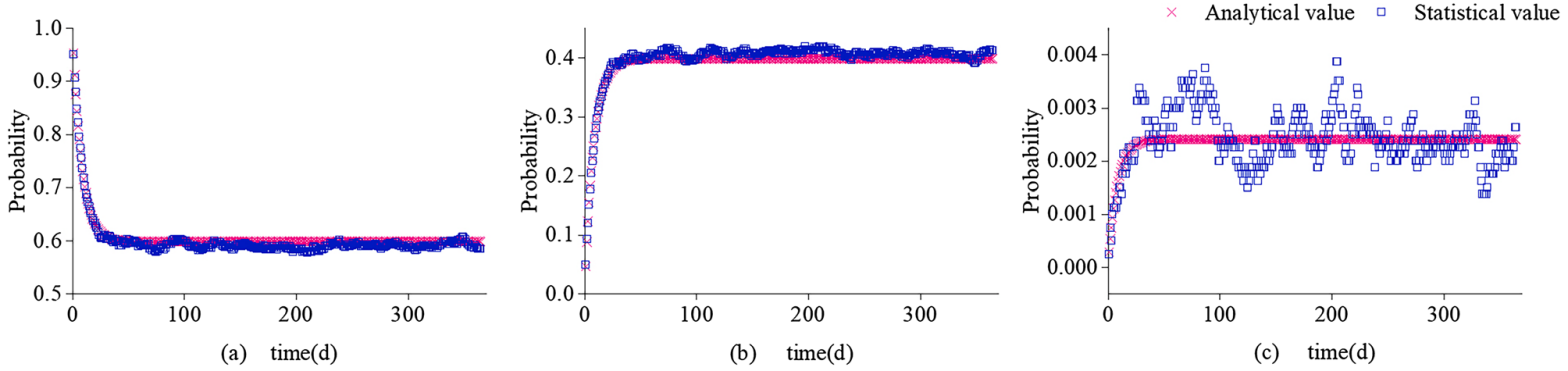
Comparison of statistical and analytical values. (**a**) Probability of being at normal, (**b**) Probability of being at risk, (**c**) Probability of being at fault.

Correspondingly, the system times (*m*_*ij*_) in various states from the 1st to the 365th time interval over 8000 simulations were respectively counted. Some of the statistical results are shown in Table 4. The statistical value ${\varvec{\phi}}^{{^{\prime}}} (1) - {\varvec{\phi}}^{{^{\prime}}} (365)$ corresponding to the analytical value ${\varvec{\phi}}(1) - {\varvec{\phi}}(365)$ can be obtained by calculating $m_{ij} /8000$. A comparison of the element values corresponding to the values ${\varvec{\phi}}(1) - {\varvec{\phi}}(365)$ is shown in Fig. 6. Note that the analytical value and statistical value change trend in the 365 time sections is basically the same.

**Table 4:** System states statistics.

Time	System states samples			${\varvec{\phi}}^{{^{\prime}}} (1) - {\varvec{\phi}}^{{^{\prime}}} (365)$			Total
	*e*_1_	*e*_2_	*e*_3_	*e*_1_	*e*_2_	*e*_3_	
*t*_1_	7608	390	2	0.951000	0.000250	0.048750	8000
*t*_2_	7252	742	6	0.906500	0.000750	0.092750	8000
…	…	…	…	…	…	…	…
*t*_365_	4679	3300	21	0.584875	0.4125	0.002625	8000

**Figure 7:**
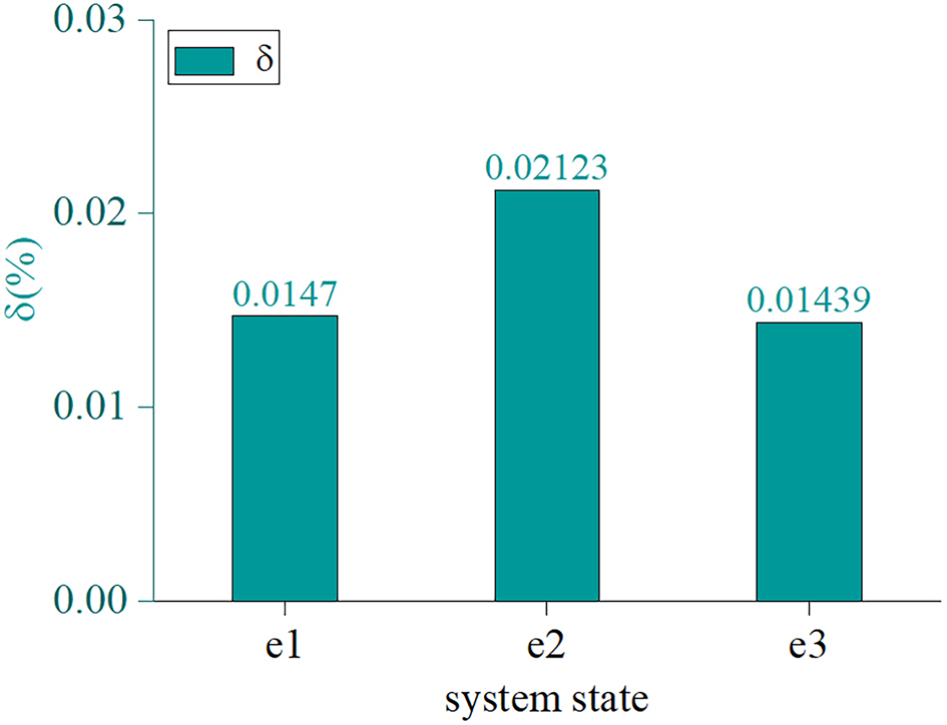
Error between statistical value and analytical value.

In order to determine whether it is effective to use the Markov chain for evaluating ventilation system reliability, a system reliability analytical value and statistical value error analysis was carried out, while the statistical value ${\varvec{\phi}}^{{^{\prime}}} (1) - {\varvec{\phi}}^{{^{\prime}}} (365)$ was regarded as the ventilation system’s true reliability in 365 time sections. Figure 6 shows that after the 50th time section, the ventilation system tends to stabilize. Over the course of 8000 simulation times, between the 50th and 365th time section, the system is in *e*_1_, *e*_2_, and *e*_3_ 1,492,394, 1,029,422, and 6184 times, respectively. The statistical value of the system's stationary state probability is calculated as $\pi_{i}^{^{\prime}} (\infty ) = [0.590346{\kern 1pt} {\kern 1pt} {\kern 1pt} {\kern 1pt} {\kern 1pt} {\kern 1pt} {\kern 1pt} {\kern 1pt} {\kern 1pt} {\kern 1pt} 0.407208{\kern 1pt} {\kern 1pt} {\kern 1pt} {\kern 1pt} {\kern 1pt} {\kern 1pt} {\kern 1pt} {\kern 1pt} {\kern 1pt} {\kern 1pt} 0.002446]$, while the stationary state’s relative error between the analytical value and the statistical value is shown in Fig. 7. The relative error of the ventilation system’s analytic value and statistical value is < 5% in all three states, which meets the error requirements.

The ventilation system’s operational state in the XQ mine was simulated 8000 times. Moreover, 365 time intervals were simulated each time, thus, a total of 2,920,000 system state time sections were obtained. State 1, state 2, and state 3 were observed 1,751,047, 1,161,933, and 7020 times, respectively; thus, state 1 > state 2 > state 3. In the actual operation process, the ventilation system is usually in the normal working state, less in the risk or fault state. The ventilation system risk state is mainly caused by roadway deformation and air door/fan aging, which is not easy to detect and check. In the long run, the quantitative change causes qualitative change and leads to the occurrence of ventilation system failure, therefore, the probability of the ventilation system being in the risk state should be between the normal state and the failure state. It can thus be demonstrated that the mine ventilation system operational process simulated by the Monte Carlo is consistent with the actual operation.

### Results analysis

Based on the results presented above, it is practical, feasible, and suitable to evaluate ventilation system reliability based on the Monte Carlo simulation method and Markov chain theory. According to the reliability evaluation results of XQ mine, the analysis is as follows:

- When the XQ mine’s ventilation system was put into operation, the probability of staying in the normal state gradually decreases, while the probability of being in the risk state and fault state gradually increases, and this period is in " Infant Mortality ". For complex systems like ventilation systems, the infant failure rate is always unexpectedly high. Therefore, the functional requirements and physical structure of the system should be fully considered in the design of the ventilation system.
- As time increases, the operating state of the system gradually stabilizes, and this period is in "Random Failures". This period is the optimal working period of the ventilation system, which is the focus of our attention and the validity period for evaluating the reliability of the system.
- In the later stages of system use, the failure rate increases due to wear and tear, fatigue, aging, and corrosion of equipment parts, etc. Therefore, if the overhaul is carried out at the beginning of "Wearout", the failure rate can be reduced economically and effectively.
- After XQ mine reaches the stationary state, the probability of being in the normal state, risk state, and fault state is 59.03%, 40.72% and 0.24% respectively. Thus, the probability of the XQ mine’s ventilation system being in the risk state is relatively high. In future production process, the roadway, fan, and air door should be regularly inspected and maintained to prevent large-scale damage and avoidable accidents.

## Conclusions

The Markov chain based ventilation system reliability evaluation method proposed herein accounts for the system’s dynamic, stochastic, and strong coupling characteristics. Furthermore, it can evaluate the ventilation system’s reliability simply and quickly, as well predict the system’s future state.

1. The ventilation system’s state transition probability matrix can be obtained, with the support of a sufficient number of ventilation system operational state samples. According to the system’s initial state and state transition probability matrix, the ventilation system’s current reliability can be quickly evaluated and used to determine the system state in the future. The ability to rapidly predict reliability is highly significant to the evaluation of ventilation system operational states, system maintenance, and planning.
2. Currently, ventilation system operational state samples are primarily obtained using the simulation method. With continuous improvement of the ventilation monitoring system digital construction level, it is expected that actual ventilation system operation samples will replace the simulation samples. Under certain accumulation conditions, a Markov model of the ventilation system operational states can be obtained in advance, so as to realize ventilation system reliability real-time analysis.
3. In this study, the ventilation system operational state was divided into three categories. However, different system operational state division results can be obtained if different division principles are used. The more detailed the state division, the clearer and more significant the guidance for safe ventilation system production. Yet, as more divisions are added, the calculation difficulty increases in parallel. Thus, determining the optimal point between the number of system state partitions and the calculation cost requires further research.
4. This investigation evaluated ventilation system reliability using some assumptions and uncertainties, such as system maintenance rate, failure rate, and system state division, etc. These factors play an important role in the system reliability evaluation process, but it is difficult to accurately evaluate how much each factor contributes to overall system reliability. Experts’ experience, published literature, and the ventilation system’s historical operation behavior were jointly used herein to make reasonable assumptions concerning these factors, and provided a powerful means for the ventilation system reliability evaluation. During actual production, the staff can adjust the corresponding parameters according to the real-life situation, and get more practical results.

## Data Availability

The datasets used and/or analysed during the current study available from the corresponding author on reasonable request.
